# Valorization of spent coffee grounds and their applications in food science

**DOI:** 10.1016/j.crfs.2025.101010

**Published:** 2025-02-24

**Authors:** Uyory Choe

**Affiliations:** Department of Food & Nutrition, Konkuk University, Chungju-si, Chungcheongbuk-do, 27478, South Korea

**Keywords:** Spent coffee grounds, Valorization, Food packaging, Bioactive compounds, Functional foods

## Abstract

Spent coffee grounds are generated in large quantities as a byproduct of coffee consumption. While often discarded as waste, spent coffee grounds still contain valuable bioactive compounds, including caffeine, chlorogenic acids, and polyphenols, along with dietary fiber, proteins, and essential minerals. Because of these nutritional properties, current research using spent coffee grounds includes fermented beverages, baked goods such as muffins and cookies, and ice cream cones. This graphical review explores the chemical composition and potential health benefits associated with spent coffee grounds. Additionally, the integration of spent coffee grounds in food products including fermented beverages and baked goods, food packaging, as well as food safety concerns, is explored. Utilizing spent coffee grounds as a functional ingredient in food not only contributes to sustainability by reducing waste but also enhances the nutritional profile of spent coffee grounds integrated products. Future research should not only focus on the effective utilization of spent coffee grounds but also address potential safety concerns, such as acrylamide formation and heavy metal contamination, to ensure food safety and consumer acceptability.

## Highlights

• Spent coffee grounds are rich in fiber, proteins, and bioactive compounds.
• Spent coffee grounds mitigate obesity and have potential benefits on the brain and gut health.
• Spent coffee grounds are used in beverages, baked goods, and biodegradable packaging.
• Utilization of spent coffee grounds helps reduce waste, methane and carbon dioxide emissions, and global warming.

## 1 Introduction

**Fig. 1:**
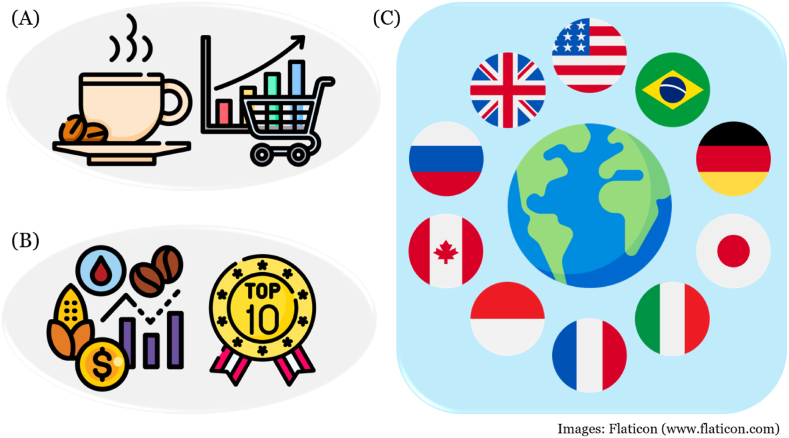
Coffee consumption and facts. (A) the trend of increase in coffee consumption; (B) coffee as one of the most traded commodities in the world; (C) top ten coffee-consuming countries.

Coffee is a beverage made by roasting the seeds of the *Coffea* plant, and it is one of the most widely consumed drinks worldwide due to its rich aroma and flavor. The caffeine in coffee has a stimulating effect, enhancing concentration, aiding in fatigue recovery, and promoting metabolism. Additionally, coffee is rich in polyphenols, which are known to provide various health benefits. Due to these reasons, coffee consumption has been increasing annually, and coffee beans are considered one of the most traded commodities globally (Samoggia and Riedel, 2019). As of 2023, the top 10 coffee-consuming countries include the United States (1788 million kg), Brazil (1332 million kg), Germany (510 million kg), Japan (450 million kg), Italy (384 million kg), France (366 million kg), Indonesia (312 million kg), Canada (300 million kg), Russia (294 million kg), and United Kingdom (222 million kg) with population size significantly influencing consumption levels (Coffee Report and Outlook) (Fig. 1).

**Fig. 2:**
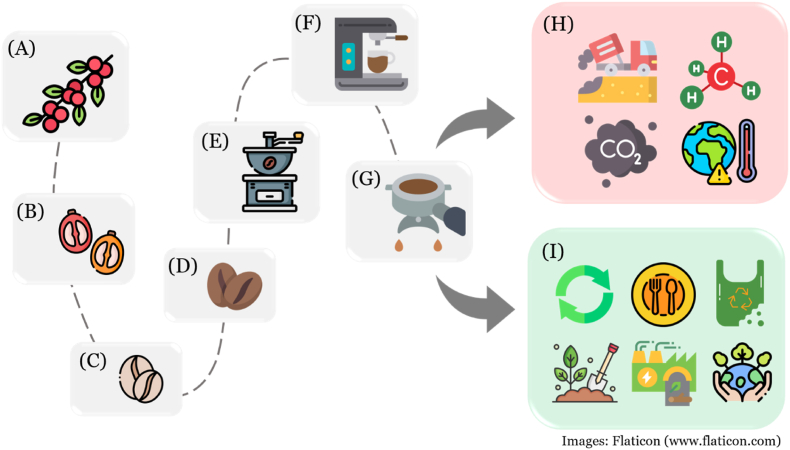
Coffee processing and two different pathways from spent coffee grounds. (A) Coffee fruits in the tree; (B) harvested coffee fruits; (C) dehulled coffee beans; (D) roasted coffee beans; (E) coffee grinding; (F) coffee brewing; (G) spent coffee grounds; (H) landfilled spent coffee grounds generating methane and causing global warming; (I) reusing spent coffee bean grounds in foods, packaging, compost, biomass to protect the environment.

Although coffee is mainly distributed as roasted beans or ground coffee, these forms have already undergone a degree of processing. To obtain coffee beans, the coffee cherry, the fruit of the *Coffea* plant, must first have its pulp removed. Each coffee cherry typically contains two seeds, which become green coffee beans after drying and de-husking. Once roasted, these beans turn brown and are then ground and extracted with hot water to produce coffee (Fig. 2).

With the high global consumption of coffee, the spent coffee grounds generated after brewing coffee are also produced in large quantities. Typically, spent coffee grounds are disposed of in landfills, where they release methane and carbon dioxide gas, a known contributor to global warming. Therefore, finding ways to repurpose spent coffee grounds as food ingredients or other applications could mitigate environmental pollution and climate change (Fig. 2).

In non-food science sectors, spent coffee grounds are being studied for applications such as fertilizer and biomass. It has been reported that spent coffee grounds can improve soil structure, aeration, and fertility, making them a good fertilizer for plants (Stylianou et al., 2018).

In addition to fertilizers, spent coffee grounds can be recycled as biomass. Among various applications, biodiesel (fatty acid methyl esters) can be produced by extracting triglycerides from spent coffee grounds using organic solvents such as hexane, followed by the addition of methanol and catalysis with NaOH or KOH. In the past, Vardon et al. successfully produced biodiesel from spent coffee grounds with a high yield of 96%, and the energy density was almost identical to that of soybean-based biodiesel (Vardon et al., 2013).

Another form of biomass is bio-oil and biochar, which is produced using defatted spent coffee grounds through pyrolysis in an oxygen-free environment. Bio-oil contains complex organic compounds such as acids, alcohols, ketones, phenols, and hydrocarbons, which can be used as fuels, chemical feedstocks, or converted into biodiesel (Vardon et al., 2013). On the other hand, biochar consists of 70–90% carbon and is utilized for soil amendment, carbon sequestration, and environmental remediation (Vardon et al., 2013).

In the field of food science, research on spent coffee grounds focuses on food products as well as food packaging. The current graphical review summarized spent coffee grounds’ chemical compositions, potential health benefits, applications, safety, and future perspectives.

## 2 Chemical compositions of spent coffee grounds

**Fig. 3:**
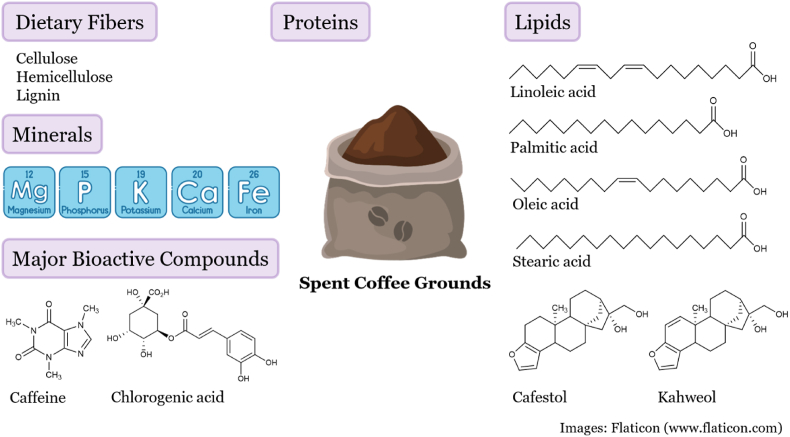
Chemical compositions of spent coffee grounds. Spent coffee grounds include dietary fibers, proteins, lipids, minerals, and bioactive compounds.

Although spent coffee grounds are a byproduct of coffee brewing, they still contain a variety of nutrients and bioactive compounds (Fig. 3). The most abundant component in spent coffee grounds is dietary fiber, accounting for nearly half of its composition. The protein content is approximately 10%, although this measurement is based on nitrogen content, which may also include nitrogen-containing compounds formed through the Maillard reaction.

Since coffee is extracted with water, lipids remain largely intact in spent coffee grounds, constituting about 2% of its composition (Ballesteros et al., 2014). The main fatty acids in spent coffee grounds are linoleic, palmitic, oleic, and stearic acids, while diterpenes such as cafestol and kahweol are also present (Vu et al., 2021; Iriondo-DeHond et al., 2019). Additionally, spent coffee grounds contain essential minerals such as magnesium, phosphorus, potassium, calcium, and iron (Pujol et al., 2013). The key bioactive compounds found in spent coffee grounds are caffeine and chlorogenic acid (Mitraka et al., 2021).

## 3 Potential health benefits of spent coffee grounds

As mentioned earlier, the primary bioactive compounds in spent coffee grounds are caffeine and chlorogenic acid. However, depending on the coffee bean variety, spent coffee grounds may also contain various polyphenols. Additionally, some portion of soluble dietary fiber in spent coffee grounds may have potential effects on gut and brain health.

**Fig. 4:**
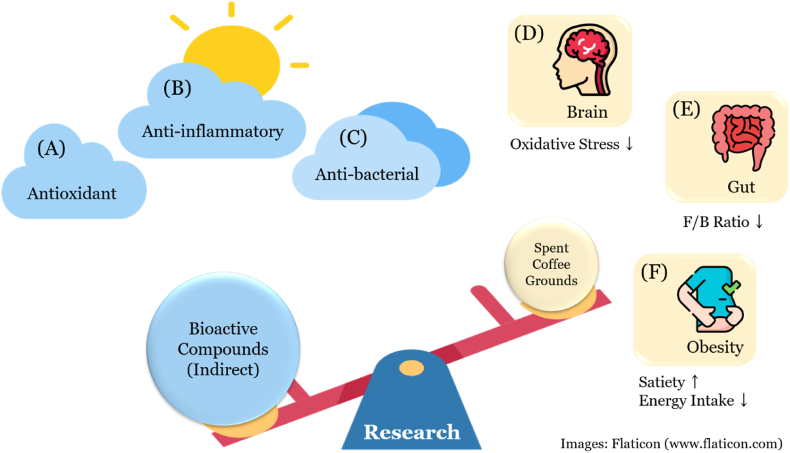
Potential health-beneficial properties of bioactive compounds from spent coffee grounds and recent potential health-beneficial research topics using spent coffee grounds. Bioactive compounds found in spent coffee grounds possess potential health-beneficial properties including (A) Antioxidant; (B) anti-inflammatory properties; (C) anti-bacterial properties. Recent potential health-beneficial research topics using spent coffee grounds include (D) protective effects on H_2_O_2_-induced oxidative stress in brain cells; (E) decreasing the F/B ratio in the gut; (F) controlling obesity by increasing satiety and reducing energy intake. F/B indicates Firmicutes/Bacteroidetes.

Nevertheless, the most significant health benefits of spent coffee grounds are expected to be derived from chlorogenic acid. Chlorogenic acid is one of the most extensively studied phenolic acids in relation to health, exhibiting antioxidant (Sato et al., 2011), anti-inflammatory (Liang and Kitts, 2015), and anti-microbial properties (Lou et al., 2011) (Fig. 4). Therefore, studies on the health benefits of spent coffee grounds often assume that spent coffee grounds will demonstrate these effects. However, actual health-related experiments using spent coffee grounds are quite rare. Recently, three interesting studies have been conducted on the health benefits of spent coffee grounds (Fig. 4).

The first study investigated how polyphenols extracted from spent coffee grounds alleviate H_2_O_2_-induced oxidative stress in *Centropomus viridis* brain cell models (Leyva-López et al., 2021). The second study examined how spent coffee grounds affect gut microbiota composition, showing that a diet containing 5% spent coffee grounds reduced the Firmicutes/Bacteroidetes ratio in mice, which is closely associated with mitigating obesity (Bhandarkar et al., 2020). The third study was a pilot study involving 10 participants, evaluating the effects of biscuits containing spent coffee grounds (Campos‐Vega et al., 2020). The results suggested that the addition of spent coffee grounds increased satiety and reduced energy intake, potentially helping to prevent obesity (Fig. 4).

To summarize, there are currently very few studies that directly assess the health benefits of spent coffee grounds. Therefore, more direct research is needed in the future.

## 4 Applications and food safety of spent coffee grounds in food science

**Fig. 5:**
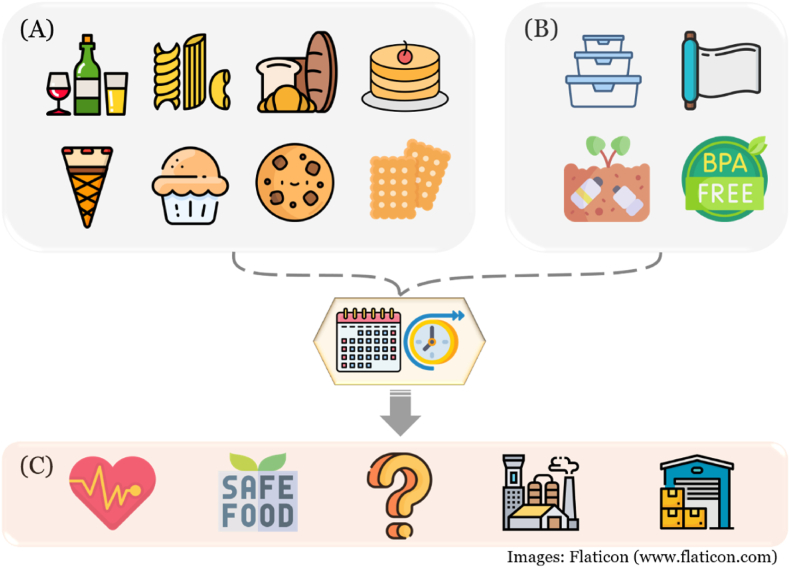
Applications of spent coffee grounds in the food science and the possible aspects of future products. (A) Food products using spent coffee grounds; (B) biodegradable and BPA-free food packaging materials using spent coffee grounds; (C) suggested future research areas of spent coffee grounds including health benefits, toxicity assessment, large-scale processing, and storage.

Due to its rich bioactive compounds and potential health benefits, spent coffee grounds have been incorporated into various food products (Fig. 5). One particularly interesting product is fermented alcoholic beverages made using spent coffee grounds, which retain coffee's distinctive flavor and aroma (Masino et al., 2022; Machado et al., 2018).

Beyond fermented beverages, spent coffee grounds have been used to develop pasta (Ahanchi et al., 2024), bread (Koay et al., 2023), cakes (Hussein et al., 2019), ice cream cones (López-Silva and García-Valle, 2024), muffins (Benincá et al., 2023), cookies (Sharma et al., 2021), and biscuits (Martinez-Saez et al., 2017). Interestingly, most spent coffee grounds-based food products have shown high acceptability among consumers. In bread research, consumer acceptability was studied for bread with 0% (control) and with 2%, 4%, 6%, 8%, and 10% spent coffee grounds. Surprisingly, the bread with 10% spent coffee grounds had the second-highest preference after the bread without spent coffee grounds (Koay et al., 2023). In contrast, for sponge cakes with 2%, 4%, and 6% spent coffee grounds, the cake with 2% spent coffee grounds was the most preferred (Hussein et al., 2019). Recently, López-Silva & García-Valle incorporated 5%, 10%, 15%, and 20% spent coffee grounds into ice cream cones and compared their sensory properties such as color, smell, taste, crispness, and general acceptability with a control. All spent coffee grounds-added ice cream cones showed no statistically significant differences compared to the control (López-Silva and García-Valle, 2024). Similarly, in cookie research, the addition of spent coffee grounds resulted in consumer preferences comparable to commercial cookies, with the rich aroma of spent coffee grounds positively influencing the cookies' taste and flavor (Sharma et al., 2021). These findings suggest that the addition of spent coffee grounds in foods does not negatively affect taste but rather contributes positively, particularly enhancing the nutritional value.

Apart from food applications, spent coffee grounds are also utilized in food packaging (Fig. 5). Recently, Dordevic et al. developed edible and biodegradable packaging using spent coffee grounds oil, which exhibited antioxidant properties and potential usability in food containers (Dordevic et al., 2023). Another study by Batista et al. explored spent coffee grounds-based biodegradable films, evaluating film biodegradability. Their findings indicated that higher crosslinking density reduced biodegradability, providing valuable insights for future research on spent coffee grounds-based packaging materials (Batista et al., 2023).

As shown above, the application of spent coffee grounds in food and packaging can offer health and functional benefits. However, safety considerations must be addressed, especially when applying spent coffee grounds to consumable food products. In the United States, if a substance is not classified as Generally Recognized As Safe (GRAS), data proving its safety must be submitted to the Food and Drug Administration (FDA) for approval. Since spent coffee grounds are not recognized as GRAS, this procedure must be followed for their use (Generally Recognized as Safe). In Europe, restrictions on the use of coffee by-products are related to their caffeine content. The European Food Safety Authority (EFSA) has established a daily caffeine intake limit of 400 mg for adults. Spent coffee grounds contain low levels of caffeine, so there are no restrictions on their use. However, during the roasting process, coffee beans are exposed to high temperatures, which can lead to the formation of acrylamide, a toxic and carcinogenic compound, through the reaction between reducing sugars and asparagine. Nonetheless, current findings indicate that acrylamide levels in spent coffee grounds are significantly below safety thresholds (Garcia-Serna et al., 2014).

Another safety concern is mycotoxins. The primary factors contributing to mycotoxin accumulation are the harvesting and post-harvest handling of coffee cherries (Napolitano et al., 2007). However, during coffee processing, these levels tend to decrease, making them unlikely to pose a significant risk. Finally, heavy metals must also be considered. Spent coffee grounds contain functional groups like carboxyl (-COOH), hydroxyl (-OH), and sulfur-containing groups (S=O), which allow them to adsorb heavy metals such as copper, lead, and cadmium in aqueous solutions. This has led to research on spent coffee grounds as an adsorbent (Davila-Guzman et al., 2016; Kim and Kim, 2020). To prevent heavy metal contamination, moisture should be removed from spent coffee grounds after coffee extraction. While processing can help prevent contamination, it is essential to verify the absence of contaminants through analytical methods. Currently, acrylamide is measured using liquid chromatography-mass spectrometry (LC-MS) and gas chromatography-mass spectrometry (GC-MS) (Wenzl et al., 2003). Mycotoxins are screened using thin-layer chromatography (TLC), LC-MS, and enzyme-linked immunosorbent assay (ELISA) (Krska et al., 2008). For heavy metals, inductively coupled plasma-mass spectrometry (ICP-MS) and atomic absorption spectroscopy (AAS) are the most widely used methods (Mukherjee et al., 2023).

To date, no safety issues have been reported regarding food products utilizing spent coffee grounds. This is likely due to the limited application of spent coffee grounds in food. In the future, to develop a wider variety of food products using spent coffee grounds, it will be essential to ensure their safety through analytical methods.

## 5 Conclusions

If effectively utilized, spent coffee grounds can contribute to human health, generate economic value, and support environmental sustainability. However, since the long-term safety of high spent coffee grounds consumption is not yet well studied, further research is needed to ensure food safety while incorporating spent coffee grounds into dietary products.

Similarly, spent coffee grounds-based food packaging is gaining attention as an edible and biodegradable material. However, further development is required before it can be commercialized on a large scale. As illustrated in Fig. 5, future research should focus on the evaluation of the health benefits of spent coffee grounds, toxicity assessment, and the feasibility of large-scale processing and storage. These efforts will pave the way for significant advancements in spent coffee grounds-based food products and packaging materials.

## Declaration of competing interest

The authors declare that they have no known competing financial interests or personal relationships that could have appeared to influence the work reported in this paper.

## Data Availability

Data will be made available on request.
